# An evaluation of Emergency Management of Severe Burn (EMSB) course in Bangladesh: a strategic direction

**DOI:** 10.1186/s41038-017-0078-8

**Published:** 2017-04-25

**Authors:** Animesh Biswas, Fazlur Rahman, Peter Maitz, Kamran Ul Baset, Jahangir Hossain, Saidur Rahman Mashreky

**Affiliations:** 1 Centre for Injury Prevention and Research, Bangladesh (CIPRB), Dhaka, Bangladesh; 2 Department of Epidemiology, Bangladesh University of Health Sciences (BUHS), Dhaka, Bangladesh; 3 The University of Sydney, Sydney, Australia

**Keywords:** Burn, EMSB, Bangladesh, Low-income countries

## Abstract

**Background:**

Burn is one of the major public health problems in Bangladesh. Specialized personnel and technologies are required, however, in many cases they are not readily available. Taking the situation into account, Interplast Australia and New Zealand, Australia & New Zealand Burn Association (ANZBA), and Centre for Injury Prevention and Research, Bangladesh (CIPRB) initiated Emergency Management of Severe Burn (EMSB) training programme for Bangladeshi physicians in 2008 to help improving their burn management skill. The study was designed to evaluate the effect of EMSB programme in Bangladesh.

**Methods:**

Both qualitative and quantitative methods were adopted. A cross-sectional survey was conducted to obtain quantitative information from 38 randomly selected EMSB-trained doctors among 380 trained physicians based on a five year database of EMSB (2008-2012). In-depth interviews (IDIs) and focus group discussion (FGD) were used as data collection techniques to get information.

**Results:**

A total of 32 participants completed the interview. It was found that 87.5% (*n*=28) doctors were using their skill in burn management that they learnt from the EMSB course. About 43.8% (*n*=14) doctors felt that the course largely helped improve their confidence. Majority (56.2%, *n*=18) of doctors stated EMSB is essential for the Bangladeshi doctors to learn better management of burns. Qualitative findings show that the courses were organized successfully with an excellent coordination, maintaining same quality and standard as running anywhere in the world. For its effectiveness, the course has been recommended to train graduate nurses and junior doctors from the periphery of the country.

**Conclusions:**

EMSB has already created a large doctor community who are able to effectively manage burn patients. It also has proven its indispensability for learning burn management skill. The EMSB established a platform to serve the burn victims and reduce the burden of injuries in Bangladesh.

## Background

Burn is a major public health problem throughout the world, especially in developing countries [1, 2]. The problem has been further compounded by the cost of burn treatment [3–5]. It requires specialized personnel and technologies that in many cases are not always readily available in many of the low-income countries [6]. In countries like Bangladesh with a population around 160 million, about 365,000 people suffer burn injuries every year, among them about 5600 people die [7]. Children are the major victims of burn injuries. Burn is the third leading cause of illness of children aged 1–4 years. About 3400 children become permanently disabled every year in Bangladesh due to burn [7]. It is a major cause of hospital admission and it requires longer hospital stay compared to any other illness, and as a result it causes a huge economic and social burden for the families [5]. The first hour of burn is crucial [8–10]. Immediate and effective assessment followed by resuscitation and timely transfer of the patient with severe burn has proven improved outcome [11]. The treatment of severe burn injury is long, painful, and costly than treating other injuries. In low-income countries, burn injuries are considered as complicated health problems as this type of medical care requires specialized staff and technologies that are expensive and not always readily available. Burn is associated with longer hospital stay, permanent disability and mental stress, and it creates an economic burden for the family as it disrupts the ability of family members to work. The number of burn incidence in Bangladesh is more than double compared to that of the developed countries [12]. It has been identified as the second biggest cause of disability in children after falls-related injuries in Bangladesh.

Aiming to help the burn injury specialists in Bangladesh better deal with burn injury patients and thus reduce burn injury-related mortality and morbidity, Interplast Australia & New Zealand and Australia & New Zealand Burn Association (ANZBA) initiated EMSB training programme for Bangladeshi physicians in 2008 in partnership with Centre for Injury Prevention and Research, Bangladesh (CIPRB) [13]. Interplast provided financial support to conduct this training in Bangladesh [10]. The course was first introduced in 1996 by ANZBA, and it was evidenced highly successful in emergency management of severe burn [14]. Between 2008 and 2012, about 529 plastic surgeons, paediatric surgeons and general surgeons received EMSB training under the project. Forty-three physicians were selected and trained so that they can better facilitate the EMSB training as instructors. Five physicians from Nepal received the training and two of them have been selected and trained as EMSB instructors. After implementation of 5 years of the training, this study was designed to evaluate the effect of EMSB course in Bangladesh.

## Methods

Both qualitative and quantitative methods were used in this study. To explore the status of knowledge and skill that the burn injury specialists received from EMSB course, a cross-sectional quantitative survey was conducted among the physicians. The 5-year database of EMSB shows that 529 doctors participated in EMSB course, out of them 417 doctors qualified the course and obtained course completion certificate. Among the 417 doctors who completed the course, a total of 37 were selected and trained as trainers of EMSB and other 380 remained as trained service providers. Among 380 trained physicians, the researchers randomly selected 10% (*n* = 38) as study participants. Finally, 32 completed the interview. A self-administered pre-tested structured instrument was utilized to collect the data from the doctors.

Qualitative design was followed to explore the perception of trainers about the course. Focus group discussion (FGD) and in-depth interview (IDI) methods were adopted in qualitative design.

For conducting EMSB course in Bangladesh, we have total 37 instructors among which six are key instructors. FGD was conducted with the six key instructors, and IDI was conducted with the seven instructors who were selected randomly from total instructor participants. Moreover, two coordinators were responsible for coordinating EMSB course in Bangladesh, and one of the faculty members from ANZBA was also interviewed in this study. Structured questionnaire was used for quantitative study while guidelines and checklist were used to obtain qualitative data. A number of probes were used to elicit appropriate information as needed. The qualitative interviews were recorded in audio tape, and some of the key points were noted in minutes. SPSS statistical software package version 21 was used to perform descriptive analysis of quantitative data. For qualitative data, each of the interviews was transcribed in local Bengali language from the audio tape. Later, transcriptions were translated into English language. Initially open code followed by selective coding of the data was performed. Data were stratified into different themes and then analysed thematically. Qualitative data were analysed manually.

## Results

### Qualitative findings

Qualitative findings were observed on participants’ characteristics who were involved in the course activities including overall course management, training methods, lesson learnt, strength and challenges of EMSB course in Bangladesh.

#### Participants’ characteristics

The key instructors in the FGD mentioned that medical graduates, especially who were involved in the surgery, plastic surgery, paediatric surgery and burn unit at specialized hospitals, were the major participants in the training. However, some of the young professionals including recently completed medical graduates were also selected for the course. The course had scope to select participants based on their qualifications and experiences in burn management in their working place. The instructors also stated that the junior doctors got opportunity to take course based on nomination given from the hospital as potential candidates. It was also found that majority of the EMSB participants were highly interested to learn from the course and were engaged in each of the session.

During discussion one of the key instructor mentioned

“EMSB committee finally selected participants for a provider course based on qualifications, experiences, seniority; and preferred the applicants who applied from the rural hospital or district hospital and want to come forward to serve the burn patients at rural community”

#### Organizing the course

The EMSB courses were organized in training venue of Bangladesh College of Physician and Surgeon (BCPS) Dhaka and at the government tertiary medical college hospitals at periphery. The courses are organized twice in a year, and in each time at least two provider courses have been organized for the medical doctors since December 2008. Standard and quality were maintained strictly in all of the courses. The course also had adequate supplies of equipment, training materials and logistics. CIPRB has played a key role in organizing this course as the hosting organization from Bangladesh. The personnel involved in the course on behalf of CIPRB have shown extreme dedication and motivation to work voluntarily. Interplast and ANZBA have provided technical and financial support. Initially, faculties from ANZBA came to Bangladesh to organize the course. In the course of time, Bangladesh EMSB committee with the help of ANZBA built their own faculty members to run the course independently. However, ANZBA has continued their monitoring visit to ensure the quality of the training. ANZBA also helped to develop key coordinators from CIPRB who played entire coordination in Bangladesh on behalf of Interplast, ANZBA and CIPRB.

“Now it is a very well-run course in Bangladesh, and same course has been running in many places worldwide. I would recommend that it shall be continued at college levels in Dhaka, or in the new facilities in Chittagong”—Representative from ANZBA mentioned.

One of the key instructors mentioned in FGD

“Our volunteers who were working as simulated patients found so devoted. They were lying down in the bed as burnt patient for about three to four hours and trainee participants got scope to practice by seeing the simulated patient, which is an excellent management for the entire course”.

Representative from ANZBA stated

“The success of running this course and the long term management of the course in Bangladesh are to be credited to the CIPRB team and the key instructors in Bangladesh. As you don’t have nurses at the level of coordinator, as a result the level of independence and success would not have been achieved without the great CIPRB team”.

#### Teaching methods

Instructors in the FGDs and IDIs mentioned that the course is following the same modules that are used in other countries where EMSB is conducted. The module has been developed by ANZBA and instructors are following the international course guideline and manual to run the course.

One of the key instructors described

“We are committed to maintain an international level standard for this very course. The teaching method is similar to any other country where EMSB is running”

Another instructor illustrated

“Our faculty members are working dedicatedly, we try to follow all training materials to be used properly. Especially in skill stations and interactive discussion, we try to interact mostly with each of the participants to make them profoundly clear with the subject”.

“It is important to teach moulage session where participants require to answer the questions under a guided prompt, which requires to be handled more carefully during teaching”— Representative from ANZBA mentioned.

#### Strengths

According to the respondents, the EMSB course has been running since 2008 and the organizers are the key instructors and faculties from Bangladesh. The organizers ensure the best utilization of highly skilled local resources to run the course. Moreover, ANZBA has developed a pool of trained coordinators to organize and coordinate the entire course independently in Bangladesh. Furthermore, the course has provided opportunity to the Bangladeshi doctors to attend the international-level course at free of cost. They believe EMSB has established a platform for doctors form different settings where they can exchange their views and experience in burn management. It also provides opportunities for regional doctors to attend the course. Five doctors from Nepal completed EMSB course in Bangladesh, and three of them have qualified as instructors. Besides, key instructors from Bangladesh have also participated in the same course in Nepal to train the Nepalese doctors.

“EMSB has maintained the standard of the course; and similar standardized format of the course is taught anywhere in the world.”—Participant from ANZBA stated.

Another key instructor spoke

“It’s a very organized course; EMSB committee in Bangladesh is working hard to maintain its quality and standard. Doctors in any faculties such as plastic surgeon, general surgeon, pediatrics surgeon, and practitioner at any position can get benefited from the course.”

Another instructor mentioned

“EMSB has a unique strength; we have number of instructors including key instructors who are most renowned and senior professors in burn and plastic surgery”.

Participant from ANZBA described

“EMSB has changed burn care in the first 24 hours in Bangladesh; the team has achieved a certain level of ownership over the course and is very effective in getting the word across. Several instructors have also helped in running courses outside of mainstream Bangladesh and internationally. This was an aim of ANZBA that you would be able to be a leading host and support in the region”.

#### Lesson learnt

Bangladesh has gathered effective lessons from the EMSB course. The EMSB committee, which consists of the professor of burn and plastic surgery in the chair, other five key instructors and key coordinators from CIPRB, is a recognized platform for the burn and plastic surgery professionals to work on burn management in Bangladesh. It has established an excellent example of practicing such international courses in other low-income country settings. This course is run by a group of highly skilled and dedicated instructors who are voluntarily contributing to the course. It has well-organized mechanisms to run throughout the year. Most importantly, CIPRB has such capacity and skill to run the course in Bangladesh with the support of ANZBA and Interplast (Table 1).

**Table 1:** Key lesson learnt

• EMSB is running by a technical committee—EMSB committee, Bangladesh since 2008 to 2012.
• Highly skill and dedicated key instructors, instructors, coordinators, volunteers are involved in the course and providing their time voluntarily.
• Independently run by the Bangladesh faculties including key instructors and other instructors.
• Bangladesh has strong capacity to run the course in country and also faculties can contribute to run the course in neighbored countries.
• National Technical organization, CIPRB is highly capable to organize and coordinate the course.
• It can be possible to run the course at central level in Dhaka and also in periphery.
• EMSB brings the scope for the doctors from primary to tertiary centre to do the course.
• Doctors at the primary centre and also in secondary centre used ESMB skill in burn management and refer patients in referral centre.
• EMSB can run in low cost and is feasible to make it sustainable.

One of the key instructors mentioned

“It is a great experience to run EMSB successfully for five years in Bangladesh; we are now capable to run the course anywhere around the country and abroad”.

Another key instructor spoke

“EMSB committee in Bangladesh acts as power house, committee sits at least twice in a year, especially before the course and play strong role in maintaining quality and standard of the course”.

“CIPRB and the fantastic team involved here have helped spread the course throughout Bangladesh; and keeping the course as same as it has been taught here; and not making any mistake or change without the knowledge of ANZBA”—participant from ANZBA described.

“EMSB also helped our doctors working in burn unit at Dhaka medical college hospital. Around nine EMSB trained doctors have managed a terrible national burn disaster that occurred at Nimotoli, Dhaka because of acquiring skills from EMSB course”-One key instructor described who is also working as head of the department in burn unit in Dhaka medical college hospital.

#### Challenges

While running the course in Bangladesh, the committee has gained significant experiences in the last 5 years. However, it has identified a number of challenges, which need to be addressed to further improve the quality of the course. To name a few, EMSB still have a limited scope for junior doctors, especially for those who have just completed Bachelor of Medicine and Bachelor of Surgery (MBBS), as a large number of experienced doctors who have previously handled burn patients are selected mostly. Another challenge is that EMSB courses are not yet possible to organize for the nurses in Bangladesh though it is of crucial importance. The instructors also argued that the course is required to be disseminated through different sources so that doctors from the periphery can participate in the course at free of cost. However, sustainability of the course is an issue. Instructors have also mentioned to explore the way-out for self-funding sources in Bangladesh so that it could be run when the donor’s fund is unavailable (Table 2).

**Table 2:** Major challenges need to be worked on

• Conduction of course at periphery hospital.	
• Involvement of junior doctors from primary health care centre.	
• Increase the number of trained instructors.	
• Media coverage and dissemination in the country.	
• Financial support to organize the course. • Sustainability in coming future.	
• Continue technical support of ANZBA	

One of the key Instructors mentioned during FGD

“It’s challenging to run the course in periphery; still we have most of the instructors from Dhaka, few from Chittagong. Therefore, we can only conduct course periphery in Chittagong medical college hospital but in other places it is difficult to get enough faculties”.

However, one of the key instructors mentioned

“It’s essential to increase number of faculties in Bangladesh because some of the facilities are now being migrated to other countries and also need to increase number of faculties from periphery hospitals”.

#### Recommendations

The EMSB course in Bangladesh has been using the module and curriculum provided by ANZBA. Epidemiologic studies, case reports and photographic materials used in this course are from Australian database. The participants in the evaluation strongly recommended using of local epidemiological data and the case reports in the course materials (Table 3).

**Table 3:** Key recommendations

• Modification in course content incorporating Bangladesh context.	
• Expand the number of courses from the central level up to periphery.	
• Organize more instructor course, refreshers course for already trained instructors.	
• Require to organize course for nurses.	
• Work on sustainability.	

One of the key instructors mentioned

“We need to revise the EMSB provider’s manual including power point slides and incorporate Bangladeshi epidemiology and also different burn cases from the country context”.

### Quantitative findings

Of all the 529 doctors participated in the EMSB course, majority (47.4%, *n*=251) came from government and private medical college hospitals while 15.3% (*n*=81) came from specialized hospitals and 12.8% (*n*=68) from the burn and plastic surgery of Dhaka medical college hospital. It was found that only 9.6% (*n*=51) doctors came from the primary health care centre, Upazila Health Complex and another 7.2% (*n*=38) doctors from secondary facility at the district level from district hospitals, 7.6% (*n*=40) from other places including medical university or private clinic.

**Fig. 1:**
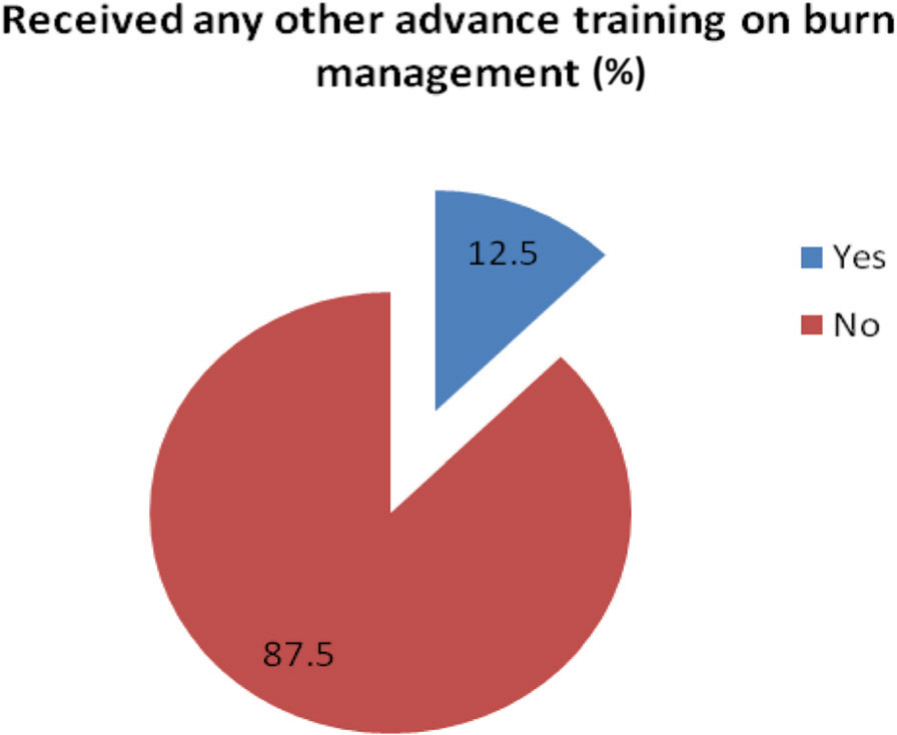
Participants received any other advanced training on burn management

**Fig. 2:**
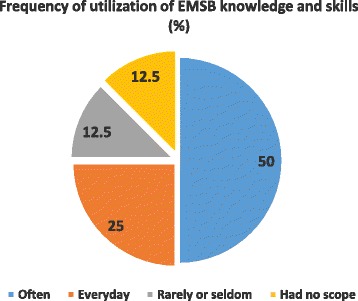
Frequency of utilization of knowledge and skill in burn patient management

Of the 32 participants completed the interview, most of them (87.5%, *n*=28) did not have any other advanced training on burn management other than EMSB (Fig. 1). EMSB course helped the doctors treat burn patients often in their facility found in 50% (*n*=16) cases, whereas 25% (*n*=8) doctors mentioned that they used their knowledge and skill daily during managing burn patients (Fig. 2). A large number of participants mentioned that EMSB improved their management skill to handle burn patient (56.2%), nearly 44% participants reported that EMSB increased their confidence level (Table 4).

**Table 4:** How EMSB course helps participants in managing burn patients

Responses (*Multiple responses)	Number of the participants (*n*)	Percentage (%)
Improved management skill	18	56.2
Increased confidence level	14	43.8
Help to handle critical situation	10	31.2
Strengthen referral system	8	25.0
Serve patient to decrease severity of burn	4	12.5
All above	12	37.5

## Discussions

Key instructors of EMSB course in Bangladesh expressed their high stratification over the overall environment of the training, they felt confident to maintain the international standard. Faculties working in EMSB course dedicating their time voluntarily. Moreover, the hosting organization in Bangladesh has been playing a vital role in organizing the course successfully. Furthermore, ANZBA also expressed their stratification over course implementation process.

Participants of the course obtained adequate knowledge and skill on emergency management of burns through EMSB, and the course also increased their confidence, managerial skills and ability to handle critical situations. Majority of the doctors trained on EMSB are using their skills in managing burn patients in their centre and referring patients properly. A study described on outcome of EMSB training which was effective to achieve better knowledge on emergency management of burns for the medical staff members [15]. Another study has shown that EMSB training has provided a remarkably positive impact in burn management during 2010 in Bangladesh in where doctors trained in EMSB played an important role in managing burn victims in one of the worst burn disasters [11]. In Bangladesh, majority of burn patients are from rural areas. For the burn injury cases in rural areas, the major challenge is the time reaching to the referral centre. For burn injuries, the patients require immediate management and proper referral to the higher centres in the right time. In the past five years, EMSB has provided opportunities for the doctors who mostly deal with burn patients in higher centres; due to minimum number of courses in a year, not enough space were available for the rural doctors. EMSB is mostly organized at the central level in Dhaka, which is also needed to expand more at the district level so that doctors from that area can also attend the course. Besides, no nurses got any opportunity to attend the course during the past five years. Thereby, courses need to be organized for the nurses too.

The course in Bangladesh showed a significant contribution in capacity development and service improvement for the burn victims. It also created demands for a wide scale-up considering wide coverage at the periphery. EMSB created demand for the doctors who are contributing in burn patients’ management. EMSB in Bangladesh needs modification in course content and teaching materials considering Bangladeshi burn scenario. The course also urged to incorporate Bangladesh chapter and epidemiology in the training manual that will be more useful and will have its practical implication.

## Conclusions

EMSB in Bangladesh has its potentiality to create a space for good number of doctors to be trained on immediate burn patient management. Doctors trained in EMSB course have already served a number of patients effectively using their knowledge and skills through EMSB. The course already created demand for doctors in learning knowledge and skills on severe burn management. Key instructors and other faculties in Bangladesh are the major strengths through an articulated and effective coordination to run the course independently in Bangladesh and maintain its quality. However, it is necessary to scale up the EMSB course further for the nurses and doctors who are working at the periphery so that majority of the burn cases can be managed immediately after an incident. Following emergency management and timely referral from periphery to the specialized centre can reduce burden of the disease and mortality due to burn injury in Bangladesh.
